# Portacaval Shunt for Portal Hypertensive Gastropathy

**DOI:** 10.1155/1997/23638

**Published:** 1997

**Authors:** John Craig Collins, I. James Sarfeh

**Affiliations:** Veterans Affairs Hospital Department of Surgery University of California Irvine 101 The City Drive Orange California 92668-9969 USA

## Abstract

Portal hypertensive gastropathy is a vascular disorder of the gastric mucosa distinguished by ectasia of the mucosal capillaries and submucosal veins without inflammation. During 1988 to 1993, 12 patients with biopsyproven cirrhosis (10 alcoholic, 2 posthepatitic) were evaluated and treated prospectively by portacaval shunt for active bleeding from severe portal hypertensive gastropathy. Eleven patients had been hospitalized for bleeding three to nine times previously, and one was bleeding uncontrollably for the first time. Requirement for blood transfusions ranged from 11 to 39 units cumulatively, of which 8 to 30 units were required specifically to replace blood lost from portal hypertensive gastropathy. Admission findings were ascites in 9 patients, jaundice in 8, severe muscle wasting in 10, hyperdynamic state in 9. Child's risk class was C in 7, B in 4, A in 1. Ten of the 12 patients had previously received repetitive endoscopic sclerotherapy for esophageal varices, which has been reported to precipitate portal hypertensive gastropathy. Eight patients had failed propranolol therapy for bleeding. Portacaval shunt was performed emergently in 11 patients and electively in 1, and permanently stopped bleeding in all by reducing the mean portal vein-inferior vena cava pressure gradient from 251 to 16 mm saline. There were no operative deaths, and two unrelated late deaths after 13 and 24 months. During 1 to 6.75 years of followup, all shunts remained patent by ultrasonography, the gastric mucosa reverted to normal On serial endoscopy, and there was no gastrointestinal bleeding. Recurrent portal-systemic encephalopathy developed in only 8% of patients. Quality of life was generally good. It is concluded that portacaval shunt provides definitive treatment of bleeding portal hypertensive gastropathy by eliminating the underlying cause, and makes possible prolonged survival with an acceptable quality of life.


*HPB Surgery*, 1997, Vol. 10, pp. 333-345
Reprints available directly from the publisher
Photocopying permitted by license only

© 1997 OPA (Overseas Publishers Association)
Amsterdam B.V. Published in The Netherlands
by Harwood Academic Publishers
Printed in India

**HPB INTERNATIONAL**

**EDITORIAL & ABSTRACTING SERVICE**

JOHN TERBLANCHE EDITOR
Department of Surgery,
Medical School · Observatory 7925
Cape Town · South Africa
Telephone: 27-21-406-6204
Telefax 27-21-448-6461
E-mail: jterblan@uctgsh1.uct.ac.za

### Portacaval Shunt for Portal Hypertensive Gastropathy

### ABSTRACT

*Orloff, M. J., Orloff, M. S., Orloff, S. L. and Haynes, K. S. (1995) Treatment of bleeding from portal hypertensive gastropathy by portacaval shunt. Hepatology; 21, 1011–1017.*

**Portal hypertensive gastropathy is a vascular disorder of the gastric mucosa distinguished by ectasia of the mucosal capillaries and submucosal veins without inflammation. During 1988 to 1993, 12 patients with biopsyproven cirrhosis (10 alcoholic, 2 posthepatitic) were evaluated and treated prospectively by portacaval shunt for active bleeding from severe portal hypertensive gastropathy. Eleven patients had been hospitalized for bleeding three to nine times previously, and one was bleeding uncontrollably for the first time. Requirement for blood transfusions ranged from 11 to 39 units cumulatively, of which 8 to 30 units were required specifically to replace blood lost from portal hypertensive gastropathy. Admission findings were ascites in 9 patients, jaundice in 8, severe muscle wasting in 10, hyperdynamic state in 9. Child's risk class was C in 7, B in 4, A in 1. Ten of the 12 patients had previously received repetitive endoscopic sclerotherapy for esophageal varices, which has been reported to precipitate portal hypertensive gastropathy. Eight patients had failed propranolol therapy for bleeding. Portacaval shunt was performed emergently in 11 patients and electively in 1, and permanently stopped bleeding in all by reducing the mean portal vein-inferior vena cava pressure gradient from 251 to 16 mm saline. There were no operative deaths, and two unrelated late deaths after 13 and 24 months. During 1 to 6.75 years of follow-up, all shunts remained patent by ultrasonography, the gastric mucosa reverted to normal on serial endoscopy, and there was no gastrointestinal bleeding. Recurrent portal-systemic encephalopathy developed in only 8% of patients. Quality of life was generally good. It is concluded that portacaval shunt provides definitive treatment of bleeding portal hypertensive gastropathy by eliminating the underlying cause, and makes possible prolonged survival with an acceptable quality of life. (Hepatology 1995; 21, 1011–1017.)**

*Keywords:* Portacaval shunt, portal hypertensive gastropathy

### PAPER DISCUSSION

In this paper, the senior Orloff and colleagues propose an additional indication for their preferred surgical operation, the total portacaval shunt [1]. Portal hypertensive gastropathy (PHG), as Orloff points out, is a recently-characterized lesion that may result in significant (albeit usually not massive) upper gastrointestinal hemorrhage [2–4]. Orloff *et al.* accrued 12 patients over a 5 year period with PHG associated with bleeding that was deemed severe enough to warrant operative intervention [1].

The pathophysiolgy of PHG is distinct from that of gastritis [2], although the earliest reports of portal decompression to treat PHG referred to it as "erosive gastritis" or "hemorrhagic gastritis" for want of a better term [5-7]. Subsequent investigations have shown that the pathogenesis of PHG is a consequence of adverse events in the gastric mucosal microvasculature [8-10]. Gastritis, by contrast, is primarily an inflammatory phenomenon [2].

Ongoing work at the basic science level continues to elucidate the functional and structural features of the portal hypertensive gastric mucosa that result in PHG [8-12]. Animal models of PHG and a few preliminary studies in humans have demonstrated that submucosal edema, venous ectasia and hypo-oxygenation of the gastric mucosal surface are observed consistently. An important additional feature is gastric mucosal microvasculopathy, characterized by hypertrophy of capillary endothelial cells with diminished cross-sectional area of the capillary lumen, which may account for the local hypo-oxygenation and compromised defensive barrier function. The latter is characterized by increased back-diffusion of hydrogen ions and increased susceptibility to damage by noxious agents such as alcohol and aspirin [12].

A recent consensus conference sponsored by the New Italian Endoscopic Club (NIEC) concluded that PHG is a clinically significant lesion, though more commonly associated with chronic than acute blood loss [13]. Propranolol and other nonselective beta-adrenergic antagonists were suggested as a means to diminish the probability of bleeding from PHG. Surgical portacaval shunts, especially total shunts (as in Orloff's series) were considered to be indicated for managing refractory disease.

In the present study, Orloff points out that endoscopic sclerotherapy of esophageal varices has been observed to precipitate PHG or make it worse [1,3]. Presumably, other methods that do not act to reduce intragastric venous pressure, such as endoscopic or surgical variceal ligation, will suffer from the same limitation. The substitution of congested gastric mucosal microvessels for congested esophageal macrovessels was an unanticipated complication of ablative treatments that has only recently come to light as PHG was recognized as a distinct clinical entity [3].

To focus excessively on either the indication or the specifics of the operation, though, may be to miss the point. Orloff has another message: when a patient presents with life-threatening hemorrhage, speed matters. Orloff's clinical practice setting represents a remarkable and unique situation. At the University of California, San Diego (UCSD), Orloff and his team take immediate control of all patients with portal hypertensive upper gastrointestinal bleeding. Within eight hours [1], such patients undergo definitive surgical decompression of the portal circulation with consequent cessation of hemorrhage. Implicit in this scheme is that patients also are afforded prompt, definitive airway control, ongoing fluid resuscitation, correction of coagulopathy, and maintenance of normothermia. In an editorial accompanying Orloff's paper, Conn [14] suggests that the speed with which the UCSD team addresses the physiologic derangement of severe portal hypertensive bleeding may be the most significant aspect of treatment. We agree.

While other workers in the field of portal hypertension advocate a variety of emerging, less invasive procedures such as variceal ligation and TIPS, Orloff's contrarian approach is to continue to find new indications for a time-tested operation [1]. As he expands his long-running clinical series from bleeding esophageal varices to bleeding portal hypertensive gastropathy, the consistency of his phenomenal clinical success is somehow reassuring.

### *References*

[1] Orloff, M. J., Orloff, M. S., Orloff, S. L. and Haynes, K. S. (1995). Treatment of bleeding from portal hypertensive gastropathy by portacaval shunt. *Hepatology*, **21**, 1011–1017.
[2] Mcormack, T. T., Sims, J. and Eyre-Brook, I. *et al.* (1985). Gastric lesions in portal hypertension: Inflammatory gastritis or congestive gastropathy? *Gut*, **26**, 1226–1232.
[3] D'Amico, G., Montalbano, L. and Traina, M. *et al.* (1990). Natural history of congestive gastropathy in cirrhosis. *Gastroenterology*, **99**, 1558–1564.
[4] Sarfeh, I. J. and Tarnawski, A. (1992). Portal hypertensive gastropathy. *Problems in General Surgery*, **9**, 431–435.
[5] Sarfeh, I. J., Tabak, C., Eugene, J. and Juler, G. L. (1981). Clinical significance of erosive gastritis in patients with alcoholic liver disease and upper gastrointestinal hemorrhage. *Ann. Surg.*, **194**, 149–151.
[6] Sarfeh, I. J., Juler, G. L., Stemmer, E. A. and Mason, G. R. (1982). Results of surgical management of hemorrhagic gastritis in patients with gastroesophaageal varices. *Surg. Gynecol. Obstet.*, **155**, 167–170
[7] Babb, R. R. and Mitchell, R. L. (1988). Persistent hemorrhagic gastritis in a patient with portal hypertension and esophagogastric varices: The role of portal decompressive surgery. *Am. J. Gastroenterol.*, **83**, 777–779.
[8] Hashizumi, M., Tanaka, K. and Inokuchi, K. (1983). Morphology of gastric microcirculation in cirrhosis. *Hepatology*, **3**, 1008–1012.
[9] Tarnawski, A., Sarfeh, I. J., Bui, H. X. and Stachura, J. (1988). Microvascular abnormalities of the portal hypertensive gastric mucose. *Hepatology*, **8**, 1488–94.
[10] Ichikawa, Y., Tarnawski, A. and Sarfeh, I. J. *et al.* (1994). Distorted microangioarchitecture and inpaired angiogenesis in gastric mucose of portal hypertensive rats. *Gastroenterol*, **106**, 702–708.
[11] Sarfeh, I. J., Soliman, K. and Waxman, K. *et al.* (1989). Impaired oxygenation of gastric mucosa in portal hypertension: The basis for increased susceptibility to injur. *Dig. Dis. Sci.*, **43**, 225–228.
[12] Sarfeh, I. J. and Tarnawski, A. (1991). Increased susceptibility of the portal hypertensive gastric mucosa to damage. *J. Clin. Gastroenterol.*, **13**, (Suppl. 1): S18–S21.
[13] Spina, G. and Arcidiacono, R. [eds.] (1994). Gastric Endoscopic Features in Portal Hypertension: Proceedings of the Consensus Conference of the New Italian Endoscopic Club (NIEC), Milan, Milano: Masson S.p.A.
[14] Conn, H. O. (1995). Emergency portacaval anastomosis in portal hypertensive gastropathy: Another piece of the puzle. *Hepatology*, **21**, 1190–1192.

John Craig Collins, MD and
I James Sarfeh, MD
Veterans Affairs Hospital and
Department of Surgery
University of California,
Irvine 101 The City Drive
Orange, California 92668-9969
United States of America

### Is there a Role for Radical Surgery in Advanced Gallbladder Carcinoma?

### ABSTRACT

*Miyazaki, M., Itoh, H., Ambiru, S., Shimizu, H., Togawa, A., Gohchi, E., Nakajima, N. and Suwa, T. (1996) Radical surgery for advanced gallbladder carcinoma. British Journal of Surgery; 83, 478 – 481.*

**Forty-four patients with advanced gallbladder carcinoma (18 with stage $pT_3$ and 26 with stage $pT_4$ of the Union Internacional Contra la Cancrum classification) were aggressively managed by extended heptatic resection in 33 patients, bile duct resection in 28, pancreaticoduodenectomy in seven, gastrointestinal resection in eleven and portal vein resection and reconstruction in seven. Adjacent organ involvement was classified as follows: type I, hepatic involvement with or without gastrointestinal invasion (Ia, Ib); type II, bile duct involvement with or without gastrointestinal invasion (IIa, IIb); type III, hepatic and bile duct involvement with or without gastrointestinal invasion (IIIa, IIIb); type IV, gastrointestinal involvement without hepatic or bile duct invasion. Fourteen of 15 patients with type I tumours had a curative resection compared with seven of 26 with type III lesions ($P$< 0.0001). The surgical mortality rate was two of 15 patients with type I tumours, seven of 26 with type III tumours and nine of 44 for the whole group. The long-term survival rate after curative resection was four and two of 23 at 3 and 5 years respectively, significantly better than two and none of 21 at 1 and 2 years after**

